# A pilot investigation of quality of life and lung function following choral singing in cancer survivors and their carers

**DOI:** 10.3332/ecancer.2012.261

**Published:** 2012-07-11

**Authors:** NS Gale, S Enright, C Reagon, I Lewis, R van Deursen

**Affiliations:** 1 Department of Physiotherapy, School of Healthcare Studies, Cardiff University, Heath Park, Cardiff, CF14 4XN, UK; 2 Department of Occupational Therapy, School of Healthcare Studies, Cardiff University, Heath Park, Cardiff, CF14 4XN, UK; 3 Tenovus, Gleider House, Ty Glas Road, Cardiff, CF14 5BD, UK

## Abstract

**Background:**

The diagnosis of cancer creates a wide range of social and emotional problems to patients and carers. However, delivering effective psychological, emotional, and social support remains a challenge. This pilot study evaluated quality of life (QoL) and lung function before and after three months of choral singing in cancer survivors and their carers.

**Methods:**

At baseline, 30 cancer survivors and their carers, mean (standard deviation) age 60 (10), completed questions about QoL (SF-36), anxiety and depression, and the multidimensional fatigue score. Lung function was measured by spirometry, and respiratory musclestrength (maximal inspiratory pressure, MIP; maximal expiratory pressure, MEP) was also measured. Assessments were repeated after three months of singing in the choir, and 10 participants completed semi-structured interviews to explore their experience of the choir.

**Results:**

After three months of choral singing, 20 subjects repeated the assessments. Several domains of the SF-36 improved, including vitality, social functioning, mental health, and bodily pain. There was also a trend of reduced anxiety and depression, despite no change in fatigue. Spirometric measures of lung function were unchanged; however, there was a trend of increased MEP. Themes from the interviews revealed that the choir provided a focus, so the future participants felt uplifted and had greater confidence and self-esteem.

**Conclusions:**

This pilot project provides preliminary data which suggest choral singing may improve QoL and depression, despite no physiological change in cancer survivors and their carers. Choral groups offer a support mechanism applicable to cancer patients, carers, and supporters, and may be relevant to other chronic conditions. Further research examining the efficacy of this intervention in a larger controlled study is warranted.

## Background

The diagnosis of cancer creates a wide range of social and emotional problems, not only for the individual with cancer but also for family members and friends [1]. This can result in feelings of isolation for the person with cancer which are often compounded by the social withdrawal that can occur as a result of fatigue, depression, and demanding treatment regimes [2]. A further problem faced by many cancer patients is the lack of effective psychological and emotional social support [3]. Patients often receive a lot of support immediately post-diagnosis, with support decreasing within the first year [4].

Social support from family members and friends can help to counteract the negative effects of symptoms on quality of life (QoL) [5]. Psychosocial support groups can help to reduce the impact of the cancer with improved mood and reduced anxiety and depression [6]. Research has shown that clinical populations who participate in organised singing groups feel an increased sense of connection to their community and to each other [7] and have enhanced QoL and emotional well-being [8].

Patients with cancer frequently suffer loss of muscle mass which may also affect the respiratory muscles, with implications for breathlessness, daily activity, and QoL as seen in other patients who suffer loss of muscle mass from chronic disease [9]. In addition to the psychological benefits of singing, it is well known that respiration has a key role in generating the voice. The practice of singing involves strong and fast inspirations, followed by extended, regulated expirations. In addition, people who sing are practicing a particular type of respiratory exercise that repeatedly demands diaphragm contractions for full inspiration, followed by sustained contractions of expiratory muscles against semi-closed vocal chords during expirations [10]. Previous investigations have shown that singing can improve lung function in patients with chronic lung disease [11, 12] and QoL [13]. The effect of singing could therefore potentially improve breathing capacity in patients who have cancer- and dyspnoea-related fatigue.

It is apparent that there is a great potential for the use of voice and singing within a choir for persons with cancer and their families but the research and literature are lacking. Therefore, the aim of this pilot study was to examine changes in QoL and fatigue following the participation in a choir using a mixed methodological approach in subjects who have received treatment for cancer and their families. A secondary aim is to establish whether singing has any further benefits in improving lung function in subjects with cancer and their friends and families (carers).

## Methods

### Sing for Life Choir

Sing for Life is a community choir set-up by Tenovus in January 2010, with the aim to provide support and a focus to anyone who has experienced cancer, including family, carers and friends, as well as cancer patients. It is open to all individuals regardless of musical experience or ability, with weekly rehearsals for a minimum of 2 h. A number of concerts are arranged through the year by Tenovus, and the choir is run in partnership with the motivational and team building company Sing and Inspire who arrange music and conduct the choir rehearsals and performances.

### Study participants

Thirty individuals were recruited to the study. Participants had a previous diagnosis of cancer, of which the type and time since treatment was variable. In addition, individuals who had been affected by cancer through a partner (carer) or friend (supporters) were included. Patients currently undergoing treatment for their malignancy or individuals diagnosed with respiratory disease were excluded. Ethical approval was gained from the School of Healthcare Studies Research and Development Ethics Committee at Cardiff University.

As this was a pilot investigation, a sample size calculation was deemed unnecessary. For the qualitative phase of the study, a purposive sample of five cancer survivors and five carers was deemed appropriate for this methodology, which does not aim for full data saturation. These were individuals who had completed questionnaires before and after singing for three months. The purpose of the qualitative study was to capture the rich, individual experiences of participants, noting common themes where apparent. The questions included in the interviews are available in the Appendix.

### Questionnaires

The following questionnaires were self-completed by participants on commencement of the choir and 3 months after the first assessment.

### Quality of life

The RAND SF-36 questionnaire is a self-administered questionnaire which assesses multiple dimensions of QoL. It is a generic questionnaire which has been validated in a number of populations including cancer and can be used to compare healthy and pathological conditions [14].

### Anxiety and depression

The hospital anxiety and depression (HAD) scale is a self-rated, widely used assessment tool for anxiety and depressionin patients with both somatic and mental health problems. It has good sensitivity and specificity as other commonly used screening instruments [15].

### Fatigue

Fatigue was measured using the Multidimensional Fatigue Inventory (MFI-20), a 20-item self-report instrument. It covers the following dimensions: general fatigue, physical fatigue, mental fatigue, reduced motivation, and reduced activity. Participants indicate on a five-point scale the extent to which each particular statement applies to him or her. An equal number of items are worded in a positive and in a negative sense to counteract response tendencies. High-MFI scores indicate a high degree of fatigue [16].

### Lung function

Assessment of respiratory function included spirometry (Micro Lab (ML3500) (Micro Medical Ltd, Kent), to determine forced expiratory volume in 1 s (FEV_1_), forced vital capacity (FVC), and their ratio (FEV_1_:FVC). Maximal inspiratory static mouth pressure (MIP) and maximal expiratory static mouth pressure (MEP) were recorded (Micro lab, UK). The best of the three attempts was recorded for all tests. All assessments were undertaken with the subject in a seated position and wearing a nose clip.

### Statistical analysis

The quantitative data were analysed using SPSS (version 16, Chicago, IL). Data which were normally distributed were analysed using the independent and paired *t* tests. Differences between completers and non-completers were analysed using the independent *t* test (parametric data) and the Mann–Whitney *U* (non-parametric data). Differences before and after the choir were analysed by the paired *t* test (parametric data) and the Wilcoxon test (non-parametric data). The Kruskal–Wallis test was used to compare more than three groups of data (patients, carers, and supporters). Categorical data were compared using the *x*^2^ test (HAD score categories). The significance level was set at *P* < 0.05.

### Qualitative data

Ten individuals (seven females, three males), who participated in the choir and the quantitative analysis, were interviewed in order to gain additional information regarding the perceived effects of singing in the choir. The semi-structured interviews lasted between 10 and 51 min and were held in the participants’ homes. Questions asked included reasons for joining the choir, previous experience of singing, expectations of the choir, benefits and downsides of being in the choir, as well as physical effects. Questions for the interview were derived from themes identified in the previous research [17].

All the interviews were transcribed verbatim, and the transcripts were verified by the participants prior to analysis. Based on Interpretative Phenomenological Analysis (IPA) [18], transcripts were analysed systematically to identify themes from each participant. The analytic process was as follows:

1. Interview transcripts were read a number of times, to ensure that a general sense of the account was obtained and notes were made of potential themes.
2. Returning to the beginning, the text was reread and any emergent themes identified and organised tentatively.
3. Attention was then focused on the themes themselves to define them in more detail and establish their interrelationships.
4. The shared themes were organised to make consistent and meaningful statements which contributed to an account of the meaning and essence of the participants’ experience grounded in their own words.

## Results

Thirty individuals were recruited to the study. There was no difference in any of the domains of QoL, anxiety depression or fatigue between the patients, carers, and supporters (*P* > 0.05, Kruskal–Wallis test) at baseline.

Nineteen of the participants had no experience of singing in a choir within the last five years, eleven were current members of another choir. There was no difference in measures of pulmonary function (FEV_1_, FVC, and their ratio, or MIP or MEP) between individuals who were new to singing and those already participating in a choir. Similarly, there were no differences in the domains of QoL, anxiety, depression, or fatigue between novice and experienced choristers (*P* > 0.05).

According to the HAD scale, at baseline, six individuals had high levels of anxiety and three had high levels of depression (HAD score 11–21). Nine were on the borderline of high anxiety and five had borderline of high depression (HAD score 8–10). The remaining had normal levels of anxiety and depression (HAD score 0–7).

Twenty-three participants completed the study assessments. Reasons for non-completion were as follows: three did not continue with the choir and four missed more than four rehearsals a month prior to the final assessment. Baseline characteristics did not differ between individuals who completed study assessments and those who did not (*P* > 0.05) (Table 1).

Pre–post choir assessments were carried out in the patients who completed all assessments (*n* = 23) (Table 2). Although there was no change in spirometric measures of lung function, there was a trend showing increased MEP (Table 2).

Several domains of the SF-36 improved after three months of choral singing, including vitality, social function, mental health, and bodily pain. There were also trends showing improved emotional role (*P* = 0.083), reduced anxiety (*P* = 0.069), and depression (*P* = 0.054), and fewer individuals had high levels of anxiety compared to baseline (Table 3). None of the domains of fatigue changed after three months of singing (*P* > 0.05) (Table 4).

### Qualitative data

The responses of the individuals who took part in the qualitative interviews were examined to gain insight into their experience of singing in the choir. The following themes which emerged from the data will be discussed below: friendship and support, a common goal and focus for the future, improved mood, increased confidence and self-esteem, an extraordinary choir, physical effects, challenges, and future directions.

#### Friendship and support

Motivations for joining the choir were varied and included the need for social interaction with and support from people who had been through similar experiences. Whilst some participants stated that they had joined the choir specifically for support, others wished to provide support to other choir members and felt that their participation was a way of saying “thank you” to Tenovus (who funded the choir). Having a common cancer experience also provided participants with a connection. The choir gave them a sense of belonging and, for those who wished, an opportunity to share their experiences. This was deemed important because participants felt that people with no experience of cancer could not fully understand what they had been through.

“We’re there for the social side of it, to help people to support people, for people to know there are other people going through treatment and know how they feel, but there’s a connection there.”

Female, 61

“I think because you’ve had cancer, or you’ve looked after someone with cancer, they will say you know how I feel. You know what it’s like. So they feel they haven’t got to express how they feel, which is not always easy anyway. They take comfort from the fact that someone else knows, or has been through that experience, without word you’ve got that empathy.”

Female, 67

“They’ve all had some problems. Not always what I’ve had, but similar things, they’ve gone through similar things and you can talk to them.”

Female, 64

“They can ask me about my cancer and stuff like that, because they all know. They’ve either lost someone to cancer, they’re going through it themselves, or they know someone. It’s a place where you can talk about it or not talk about it. That’s the brilliant part of it.”

Female, 50

#### A common goal and focus for the future

Participants reflected that working towards a communal goal (for example, performing at a concert) created a sense of camaraderie which bonded the group. The choir rehearsals gave participants something to look forward to each week, and singing was viewed as a welcome distraction from the cancer. Public performances by the choir also provided a focus for the future and maintained the interest of the group.

“Doing something like singing and making mistakes together and getting it right, brought people together, and you start to make friends.”

Female, 46

“There’s lovely camaraderie there, from singing. It’s nice to have something all the family can do. It’s been good.”

Male, 60

“So it’s something to look forward to, I suppose, every week. Something different.”

Female, 61

“The gigs are vital really, without any gigs, turning up for choir practice, people would lose interest. And it brings people together and it’s something to look forward to.”

Male, 51

“There’s a lot of people that need enjoyment after cancer. It gives somebody help, to think of something else, other than their illness really.”

Female 64

#### Improved mood, increased confidence, and self-esteem

Five participants stated that singing improved their mood, lifted their spirits, and increased their levels of confidence and self-esteem. This may be related to the sense of achievement and fulfilment gained through practising and making an improvement in performance.

“It takes you out. It lifts you … it makes you feel happy singing.”

Female, 69

“You feel elated. It makes you feel uplifted. It makes you feel a lot better. And you forget that you’re there because you had cancer, really. It’s brilliant.”

Female, 64

“We’ve got a focus now, we can do something together and we sing and we enjoy it, and it really does make you feel well.”

Female, 46

“I think confidence really and self-esteem …, [I] generally feel a lot better … after joining the choir.”

Male, 51

#### Sing for Life is an extraordinary choir

Participants suggested that the Sing for Life Choir was different from traditional choirs because its purpose is as much about providing support as about singing. The choir is also open to all ages and abilities; this differs from some traditional choirs which include only specific groups of people (for example, people who pass an audition). Also there is not a need to be able to read music as choir members are given materials with which to practise between rehearsals. One participant who been singing for some years commented that although Sing for Life was a different kind of choir, it complemented her participation in a traditional choir. The choice of modern music by the Sing for Life Choir was also welcomed by participants who perceived it as more uplifting than classical music.

“This choir is not an ordinary choir. I call it an extraordinary choir.”

Female, 61

“I know my singing isn’t strong enough. I accept that, I’m happy with that. This choir is geared to allow everyoneto come in.”

Male, 51

“It’s brought a new dimension to the musical part of my life. So it’s very much complimenting [my participation in another choir], I don’t think it’s duplicating anyway, it’s a totally different choir.”

Female, 57

“It is more free and easy, it is not so rigid, you know. It’s more fun and more modern singing, and it’s lovely.”

Female, 69

#### Physical changes

Participants offered some comments to suggest that singing may improve breathing and posture and encourage physical activity. The choir gave participants a reason to get out of the house and the rehearsals involved movement which increased their activity.

“I suppose you do stand properly, you think about your posture, because you’re supposed to be opening your lungs and everything.”

Female 61

“I’m getting out and about a bit more, so I’m walking with the walker…. I wouldn’t be doing that walking if it wasn’t for the choir.”

Female, 50

#### Challenges and future directions

Two participants felt that there were some challenges to the future of the choir. These were related to the arrival of new members changing group dynamics and maintaining the connection between choristers. There was also concern that some people may join the choir simply because the concert trips are subsidised. However, the participants recognised the need to avoid the development of a closed group, which would not welcome new members. One participant who sings in another choir suggested having representatives in different sections of the choir to introduce new members and having new member sessions every quarter.

“I think for me the only downside is not knowing some of the people who just turn up.”

Male, 51

“You have to be careful that this doesn’t become an ordinary choir, when people are there just to go on their jollies.”

Female, 57

## Discussion

This pilot study showed improvements in several aspects of QoL after 3 months of choral singing, in patients who had a previous diagnosis of cancer and their friends or partners. As measured by the SF-36, mental health improved, with gains in vitality and social function. The SF-36 is generic questionnaire which has been used in a variety of populations and allows comparison been a variety of populations. Compared to normative published data, the present cohort appeared to have lower scores at baseline [14]. There was also a trend of better emotional function, physical health, and HAD with fewer individuals classified as having high anxiety after 3 months of singing.

The improved QoL is in agreement with a number of studies in patients with chronic lung disease [12, 13] and chronic pain [19]. While in a survey of 79 older people, using a bespoke questionnaire, improvements in both mental and physical health were seen after 1 year of participating in a community choir [8].

The mechanism behind these improvements is likely to be complex. The engagement in a regular pleasurable activity with individuals with the same disease or life experiences has the potential to enhance social interaction and support [11]. Since the members of a cancer support group have had similar experiences, this can become a bonding force and can create a sense of community, as shown in the section “Qualitative data” in this study.

In general, group therapy has been found to be an efficient means of providing long-term support for cancer patients and may provide a way of engaging and returning to daily activities [20]. Whether or not singing provides additional benefit is not possible to answer in this small study. Potentially, other group activities could produce similar support mechanisms. However, the qualitative data in this study suggest that individuals valued the process of working together for a performance, which gave them a future goal and a focus for the future. Therefore, choral singing may be well placed to achieve this above other types of activities.

In this study, levels of fatigue were unchanged over three months of singing in the choir. This might have been expected given the mixed group of patients and supporters, whose levels of baseline fatigue were comparable to previously published levels of fatigue in healthy individuals [16]. Although there was no significant change in pulmonary function tests, there was a trend of increased MEP. This is in keeping with data from patients with COPD which showed that patients who attended singing classes had greater MEP than patients who did not [11]. Singing stimulates the musculature associated with respiration and requires the regulation of breathing and the extension of expiration which may increase respiratory muscle strength.

Other reported physiological benefits which have been reported after singing include increased immunoglobulin (a measure of immune function), decreased cortisol, and associated emotional stress [21, 22]. A trend of increased activity has also been reported following choral singing which may have implications for participation, health promotion, and healthcare usage [23]. These were not addressed in the present study although may be a focus of a further investigation.

The themes which emerged from the qualitative interviews compliment the findings from a survey which was developed and utilised to investigate the perceived benefits of participation in a large community choir. Of the six areas included in the survey, four similar themes were apparent in the present data, namely self-confidence, emotional wellbeing, social life, and general understanding of singing and physical health [8]. Additionally, a large mixed-methods study which included 600 English choristers identified improved mood, enhanced QoL, greater happiness, stress reduction, and emotional well-being. The qualitative interviews proposed a number of generative mechanisms some of which may apply to the present study such as: feelings of happiness and raised spirits which counteract depression, a focus which blocks worry, social support and friendship, and commitment and increased activity [24]. Thus, participation in a choir appears to improve psychosocial status through a variety of mechanisms and may be a means of providing support and other benefits to patients with potentially chronic diseases. Social, emotional, physical, and spiritual benefits of singing have also been described [17].

There are several limitations to the present study. Participants had a variable cancer experience, including patients and their family and friends, with a considerable range of time since their experience; however, QoL scores did not differ between these groups. Potentially, patients who have survived cancer may be making the most of life and may have relatively good QoL. The pulmonary function tests may have been confounded as a habituation session, which was not possible and a number of individuals had previous or current experience of singing in a choir. Previous experience of singing may also affect the effects on wellbeing. Research has shown that in response to a singing lesson, amateurs reported increasing joy and elatedness (VAS), whereas professionals did not [25]. As a pilot study, the statistical power was limited, increasing the chance of a type 2 error nevertheless improvements in mental health and trends of increased pulmonary function were shown which are worthy of further study. This study was not sufficiently powered to detect differences between groups, for the quantitative or qualitative components. However, a large scale prospective study is the focus of further investigation by the present authors.

## Conclusion

This pilot project provides preliminary data which suggest that choral singing can improve QoL and depression, despite no physiological change. Qualitative findings indicated that the choir provided social support, increased confidence, and gave a focus for the future. This provides a foundation for a larger scale study investigating the benefits of choral singing.

## Figures and Tables

**Table 1:** Baseline characteristics of individuals those who completed and who did not complete study assessments

	Completer, *n* = 23	Non-completer, n = 7	*P*
Age (years)	59 (11)	64 (6)	0.277
Height (m)	1.60 (0.11)	1.65 (0.08)	0.309
Weight (kg)	77.5 (18.1)	79.5 (11.2)	0.793
FEV_1_ (l)	2.44 (0.65)	2.56 (0.57)	0.661
FEV_1_ % predicted	102 (15)	105 (7)	0.659
FVC (l)	3.08 (0.78)	3.16 (0.80)	0.797
FVC % predicted	107 (16)	105 (6)	0.832
FEV_1_/FVC	0.79 (0.06)	0.81 (0.03)	0.313
MIP	56 (27)	81 (36)	0.058
MEP	77 (36)	101 (50)	0.175
MIP (cmH_2_O)			
MEP (cmH_2_O)			
Data presented as mean (standard deviation). *BMI*: body mass index, *FEV*_1_: forced expiratory volume in 1 s, *FVC*: forced vital capacity, *MEP* : maximal expiratory pressure, *MIP* : maximal inspiratory pressure			

**Table 2:** Physiological measures pre- and post-3-months of choral singing

*n* = 20	Pre-choir	3 months	*P*
FEV1 (l)	2.41 (0.65)	2.34 (0.66)	0.069
FEV1 % predicted	103 (15)	95 (26)	0.047
FVC (l)	3.03 (0.77)	2.94 (0.76)	0.089
FVC % predicted	108 (16)	99 (23)	0.173
FEV1/ FVC	0.79 (0.06)	0.80 (0.06)	0.482
MIP (cmH2O)	56 (27)	63 (26)	0.120
MEP (cmH2O)	77 (36)	89 (39)	0.071
Data presented as mean (standard deviation). *FEV*_1_: forced expiratory volume in 1 s, *FVC*: forced vital capacity, *MEP* : maximal expiratory pressure, *MIP* : maximal inspiratory pressure			

**Table 3:** Numbers of individuals in hospital anxiety and depression (HAD) categories pre- and post-three months of choral singing

HAD score categories	Anxiety, *n*			Depression, *n*		
	Pre	Post	Difference	Pre	Post	Difference
Normal (0–7)	8	10	+2	13	17	+4
Borderline (8–10)	6	8	+2	6	2	-4
High (11–21)	6	2	-4	1	1	0
P	–	–	0.011	–	–	0.014
*n*: number of individuals in each category						

**Table 4:** Quality of life, multidimensional fatigue, anxiety and depression, pre- and post-three months of choral singing

*n* = 20	Pre-choir	3 months	*P*
Physical function	59.8 (32.7)	66.3 (29.8)	0.073
Role-physical	63.8 (44.0)	61.3 (40.1)	0.785
Bodily pain	62.1 (28.8)	72.9 (28.2)	0.010
General health	61.5 (22.7)	60.5 (24.3)	0.688
Vitality	50.0 (24.0)	60.3 (19.7)	0.001
Social function	69.4 (25.2)	77.5 (24.9)	0.050
Role-emotional	66.7 (43.3)	76.7 (39.1)	0.083
Mental health	65.0 (17.5)	73.2 (14.0)	0.003
Overall physical health	61.8 (29.2)	65.2 (26.1)	0.258
Overall mental health	62.8 (23.0)	71.9 (21.1)	0.003
General fatigue	12.1 (5.2)	11.9 (4.4)	0.794
Physical fatigue	11.6 (6.0)	10.6 (4.9)	0.180
Reduced activity	9.4 (4.5)	8.7 (4.3)	0.234
Reduced motivation	8.5 (3.6)	7.7 (3.2)	0.273
Mental fatigue	9.1 (5.0)	8.6 (4.0)	0.489
Anxiety	8.1 (4.4)	6.8 (3.0)	0.069
Depression	5.2 (3.6)	4.4 (3.3)	0.054
Data presented as mean (standard deviation)			

## Appendix: Choir interview questions

1. How did you become involved in the Tenovus Sing for Life Choir?
2. Did you have any previous experience of singing in a choir?
3. What do you feel have been the main effects of singing in either choir?
4. Can you identify any physical effects from singing in either choir?
5. How has the sense of belonging to the choir impacted upon you?
6. Have there been any downsides/difficulties of being in the choir?
7. Did you have access to any other support groups since you cancer experience?
8. Is there anything you would like to add which sums up your experience of the choir?
